# Long head of biceps as an anterior dynamic sling for recurrent anterior shoulder dislocation

**DOI:** 10.1186/s13018-025-05769-1

**Published:** 2025-04-17

**Authors:** Mohamed Hussein Khalil, Ahmed Mahmoud Gad

**Affiliations:** Cairo University, Giza, Egypt

**Keywords:** Dynamic anterior stabilization, Onlay, Glenohumeral instability

## Abstract

**Purpose:**

To evaluate the functional outcomes of arthroscopic onlay dynamic anterior stabilization (DAS) using the long head of the biceps (LHB) tendon for treatment of anterior glenohumeral instability (AGI) with limited to subcritical glenoid bone loss (GBL).

**Methods:**

Twenty-five patients underwent arthroscopic DAS using LHB tendon between March 2022 and October 2022 for treatment of anterior glenohumeral instability (AGI) with limited to subcritical glenoid bone loss (GBL) were included in a prospective study with a minimum follow-up period of 2 years. The shoulder functional outcomes were assessed using the Rowe and the Quick Disabilities of Arm, Shoulder and Hand (Quick DASH) scores both preoperative and at 2 years follow-up. Magnetic resonance imaging (MRI) was done 6 months after surgery to evaluate LHB tendon healing to the anterior glenoid.

**Results:**

The study included 25 patients complaining of recurrent AGI. Twenty-one patients were males and four patients were females. The mean age of the patients at surgery was 22.75 ± 3.24 years. The mean duration between the first shoulder dislocation episode and surgery was 5.5 ± 2.13 months. The right shoulder was injured in 15 patients while the left shoulder was involved in 10 patients. The mean follow-up period was 24.25 ± 0.82 months. DAS using the long head of the biceps tendon resulted in a statistically significant improvement of the mean Rowe and the Quick DASH scores between preoperative and 2 years postoperative. Recurrent dislocation was reported in two (8%) patients during the follow-up period.

**Conclusion:**

Arthroscopic onlay dynamic anterior stabilization using the long head of the biceps tendon is a safe and effective method for the treatment of recurrent anterior shoulder instability with GBL up to 25%.

**Level of evidence:**

Level IV, case series study.

## Introduction

Treatment of anterior glenohumeral instability (AGI) can be achieved by several operative techniques, according to whether the principal cause is a labral tear (Bankart) [1], humeral head lesion (Hill-Sachs) [2], or glenoid bone defect [3].

Traumatic anterior shoulder instability without glenoid bone loss (GBL) can be successfully treated with Bankart repair [4]. On the other hand, bony procedures such as the Bristow and Latarjet procedures offer better outcomes in cases with concomitant GBL greater than 21-25% and engaging Hill-Sachs lesions [5, 6].

Controversy still exists regarding the ideal surgical treatment for AGI with limited (0-13.5%) to subcritical (13.5-25%) GBL [7]. Bony procedures are correlated with low recurrence, but high complication rates [8]. On the contrary, clinical studies documented fewer complication rates [9], but higher recurrence rates or inadequate results for isolated or augmented Bankart repair in the context of subcritical GBL [10].

Dynamic anterior stabilization (DAS) by transfer of the long head of the biceps (LHB) tendon fills the treatment indication gap between bony and soft tissue procedures for patients with recurrent anterior shoulder instability and concomitant subcritical bone defects [11, 12]. However, DAS is contraindicated in cases of critical GBL of more than 25%, poor LHB tendon quality, and previous LHB tenodesis or tenotomy.

The present study aims to evaluate the clinical outcomes following arthroscopic DAS using LHB tendon for the management of anterior shoulder instability.

## Materials and methods

This research design is a single-center prospective study done in a tertiary trauma center between March 2022 and October 2022. The research ethical Committee approved this study protocol [(Institutional Review Board (IRB) number: N-208-2024)], and all patients signed an informed consent before joining this study. This study included 25 patients with recurrent shoulder dislocation who underwent arthroscopic DAS using LHB for treating AGI.

*The inclusion criteria were*.

1. Patients complaining of AGI, with one or more events of anterior shoulder dislocation.
2. Associated limited (< 13.5%) to subcritical (< 25%) GBL.
3. Positive anterior shoulder apprehension.

*The exclusion criteria were*

1. Associated critical GBL (≥ 25%).
2. Concomitant LHB lesion or rupture.
3. Multidirectional or voluntary shoulder instability.
4. Prior arthroscopic shoulder stabilization procedure.
5. Concomitant rotator cuff tears.
6. Acute proximal humerus fractures of the involved shoulder.

Computerized tomography (CT) with subtraction of the humeral head was used to estimate GBL preoperatively, using the method described by Barchilon et al. [13], where GBL was estimated by calculating the ratio between the depth and the radius of the best-fitted circle. The depth was represented by a line drawn from the circle center to the impaired anterior glenoid margin.

### Functional and radiological assessment

The minimum follow-up period for all patients was 2 years (range 24–26 months). The shoulder functional outcomes were assessed using the Rowe [14] and the Quick Disabilities of Arm, Shoulder, and Hand (Quick DASH) scores and compared both preoperative and at 2 years postoperative. Magnetic resonance imaging (MRI) was done at 6 months follow-up to assess the healing of the LHB tendon to the anterior glenoid.

### Statistical methods

Data were represented statistically in the form of mean ± standard deviation (± SD), range, frequencies (patient number), and percentages when suitable. A paired t-test was performed to compare numerical variables within the group. p value less than 0.05 was deemed statistically significant. All statistical analyses were performed using IBM SPSS (Statistical Package for the Social Science; IBM Corp, Armonk, NY, USA) release 22 for Microsoft Windows.

### Operation procedures

General anesthesia was administered to all patients and all surgical steps were carried out in the beach chair position. Diagnostic arthroscopy of the glenohumeral joint was first done using a standard posterior viewing portal (2 cm inferior and 2 cm medial to the posterolateral acromial edge) confirming the diagnosis of Bankart lesion with limited to subcritical GBL.

A standard anterior working portal was created in the rotator interval in an outside-in technique using an 18-gauge needle towards the middle third of the subscapularis tendon. A working cannula (Arthrex, Naples, FL, USA) with an outflow connection was positioned through the anterior portal.

**Fig. 1:**
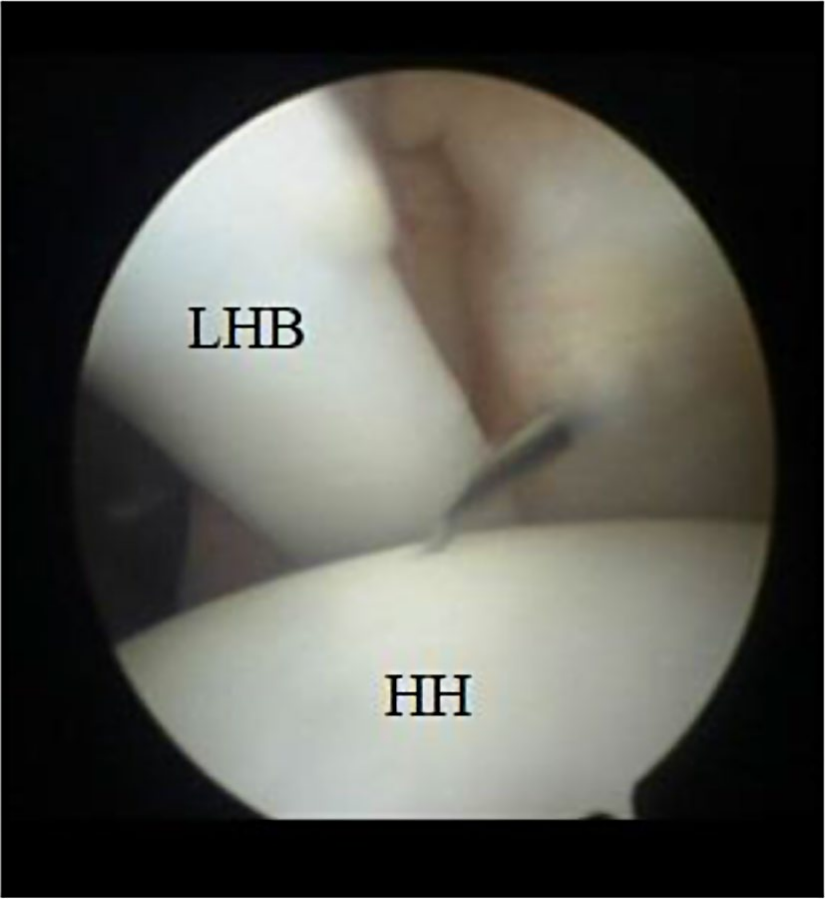
Arthroscopic imaging of the right shoulder (from the posterior portal) showing the insertion of an 18-gauge needle to establish the anterolateral portal. HH: humeral head, LHB: long head of the biceps

A third anterolateral portal was done approximately 2 cm underneath the anterolateral acromial end at the long head of.

the biceps tendon using an outside-in technique followed by the insertion of a second arthroscopic cannula (Fig. 1).

A standard remplissage procedure was done in three (12%) patients where an off-track Hill-Sachs lesion was encountered using a 5 mm anchor (Arthrex, Naples, FL, USA). Bankart repair was not performed in our study.

A cinch stitch using No. 2 fiberwire (Zimmer Biomet, Warsaw, IN, USA) was performed to secure the LHB near its glenoid attachment using a suture passer and suture limbs were retrieved through the anterior portal.

The transverse humeral ligament was incised releasing the LHB tendon down the bicipital groove using a radiofrequency probe introduced through the anterolateral portal to avoid any kinks after changing the LHB direction towards the anterior glenoid. Tenotomy of the LHB was then performed near its attachment to the superior labrum and the fiberwire suture limbs tagging the LHB tendon were retrieved from the anterior portal.

**Fig. 2:**
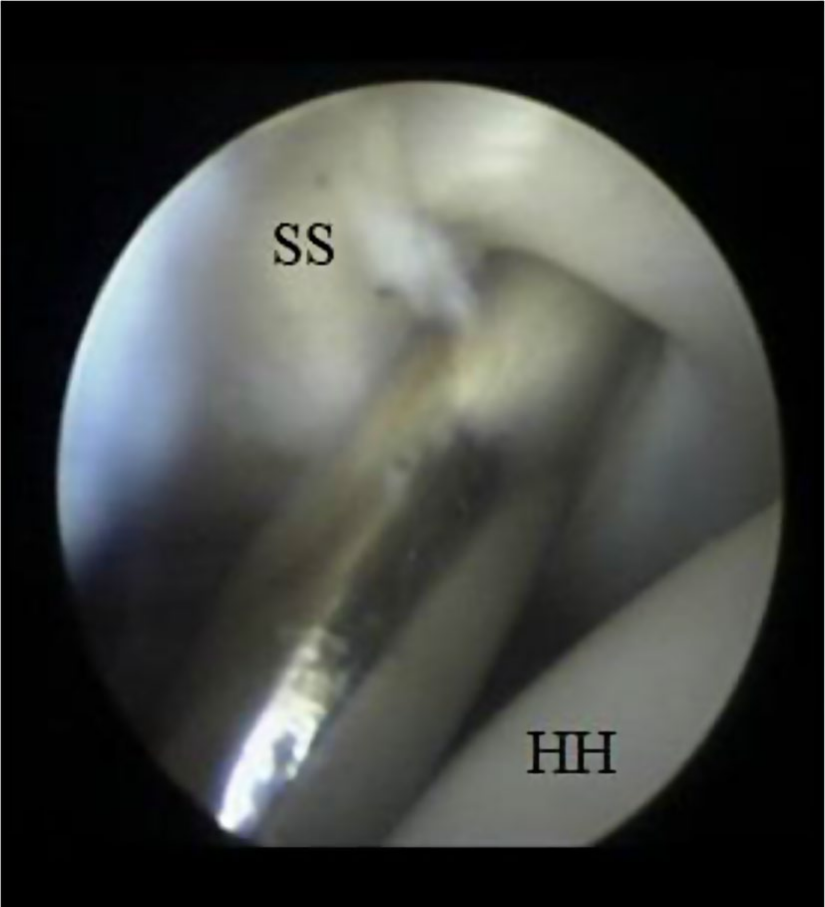
Arthroscopic imaging of the right shoulder (from the posterior portal) showing splitting of the subscapularis using a Wissinger rod introduced from the anterior portal. SS: subscapularis, HH: humeral head

**Fig. 3:**
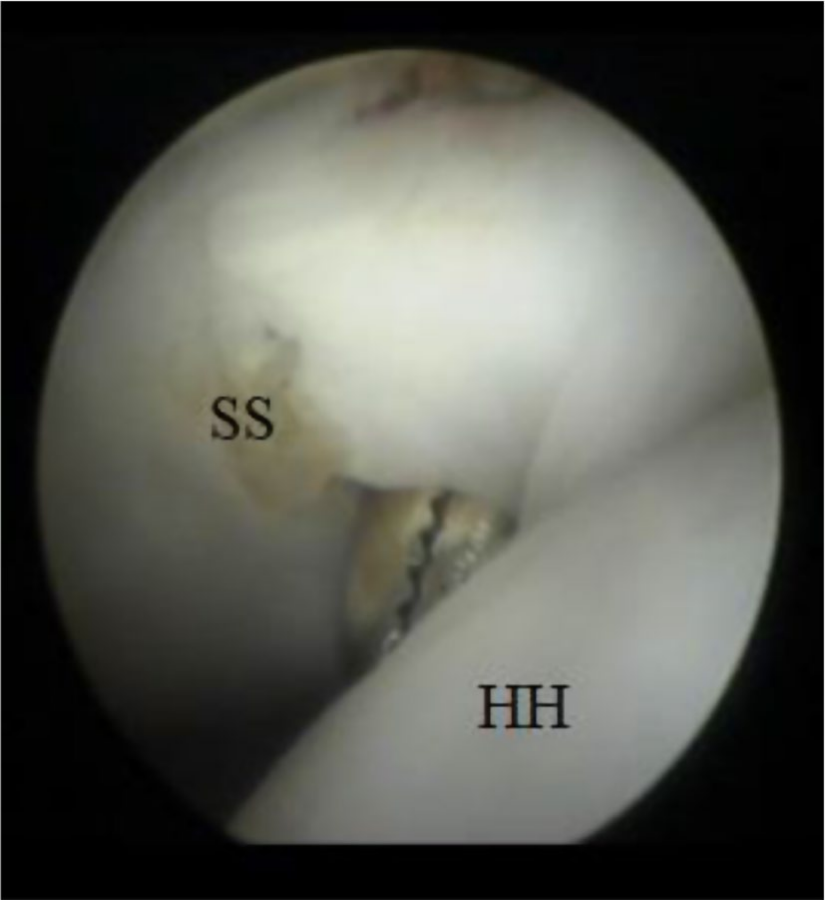
Arthroscopic imaging of the right shoulder (from the posterior portal) showing an artery forceps passed from the anterior portal to widen the subscapularis split SS: subscapularis, HH: humeral head

A horizontal split was then made using a Wissinger rod (switching stick) at the middle third of the subscapularis muscle (Fig. 2). An artery forceps was then used to widen the subscapularis horizontal split (Fig. 3).

Preparation of the anterior glenoid aspect was then performed using a 4.5-mm-wide full-radius shaver to debride the frayed labral remnants followed by rasping the anterior glenoid aspect to favor the LHB tendon healing to the anterior glenoid aspect. A bone tunnel for a 2.9 mm Quattro^®^ Link Knotless suture anchor (Zimmer Biomet, Warsaw, IN, USA) was then drilled at the 5 o’clock position (for the right shoulder) in the glenoid from the anterior portal.

**Fig. 4:**
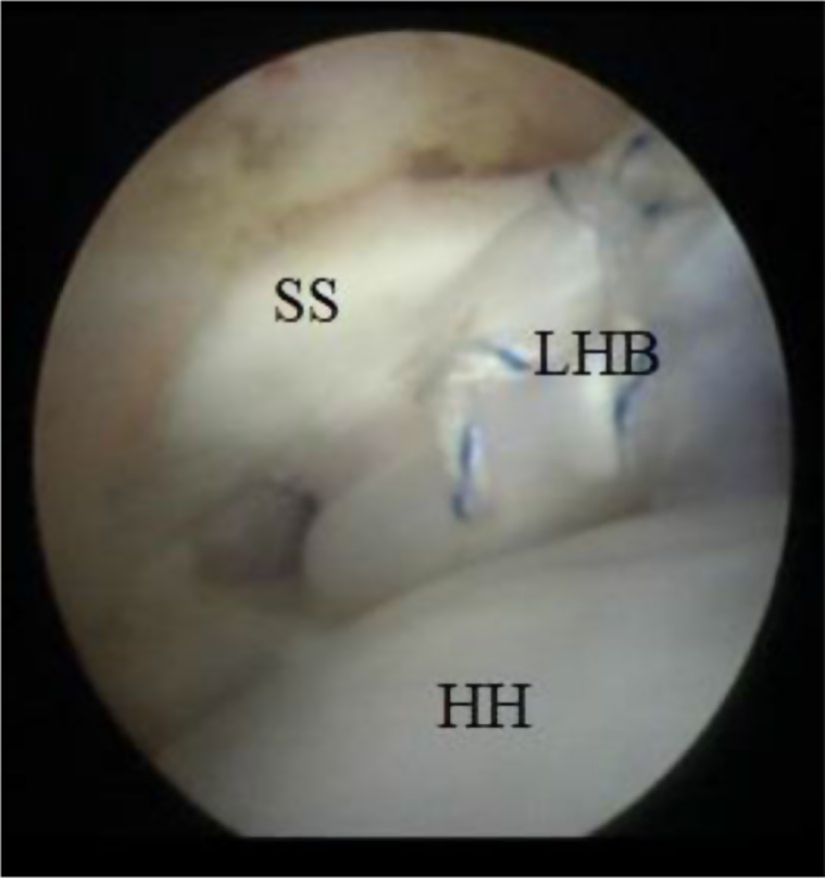
Arthroscopic imaging of the right shoulder (from the posterior portal) showing the passage of LHB through the subscapularis muscle split. SS: subscapularis, HH: humeral head, LHB: long head of the biceps

**Fig. 5:**
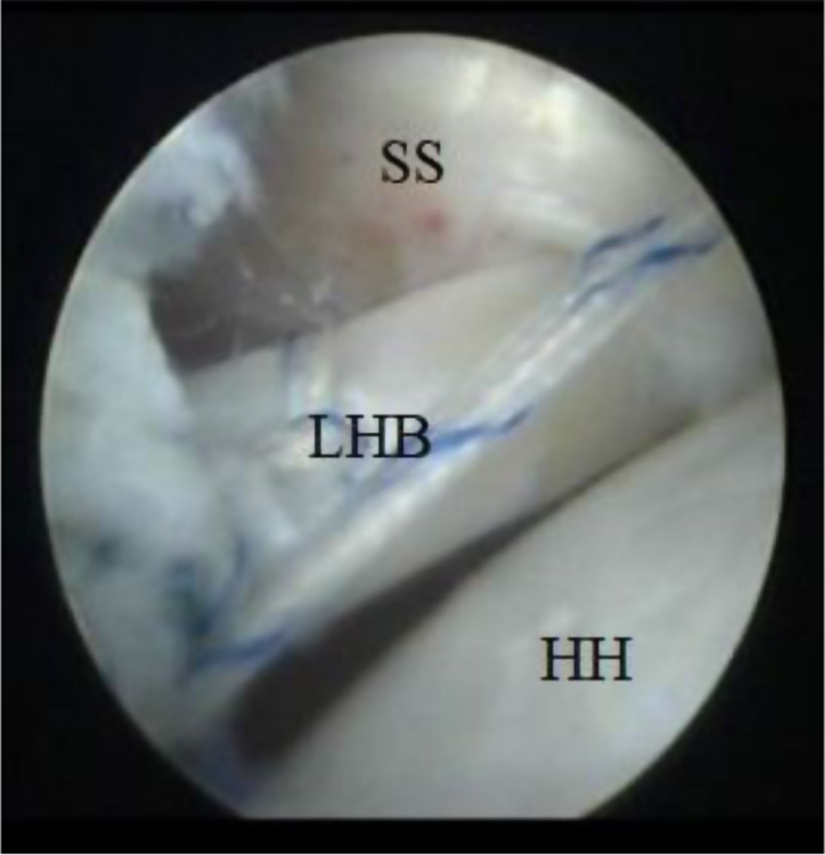
A final arthroscopic imaging (from the posterior portal) after transfer of the LHB to the anterior glenoid fixed by a knotless suture anchor. SS: subscapularis, HH: humeral head, LHB: long head of the biceps

The limbs of the LHB tendon cinch suture were passed to a 2.9 mm knotless suture anchor and the anchor was guided to the anterior glenoid socket, through the subscapularis split, maintaining tension of the fiberwire suture limbs (Fig. 4). An arthroscopic final view is shown in (Fig. 5).

## Rehabilitation

All patients followed the same rehabilitation protocol after surgery. An arm sling was used for shoulder immobilization during the first 3 weeks postoperative. Patients were encouraged to perform passive shoulder and elbow exercises as tolerated. Shoulder exercises were limited to elevation from 0° to 90°, and external-internal rotation exercises with the arm at the side from internal rotation to the neutral position. Shoulder and elbow exercises progressed from passive to active assisted exercises from 3rd to 6th week postoperative and continued for 3 months. Active resistant elbow exercises were not permitted until 6 weeks postoperative. Active shoulder abduction in external rotation was obviated until 3 months postoperative. Patients were allowed to return to sports activities 6 months following surgery.

## Results

The present study included 25 patients, who underwent arthroscopic DAS using LHB for treatment of AGI. Twenty-one patients were males, while four patients were females. The mean age at the time of surgery was 22.75 ± 3.24 years. The right shoulder was involved in 15 patients while the left shoulder was affected in 10 patients. The mean time interval between injury and surgery was 5.5 ± 2.13 months. The mean number of dislocation episodes was 7.5 ± 3.72. The mean percentage of estimated GBL was 22.5 ± 3.37. The mean follow-up period was 24.25 ± 0.82 months. All patients completed the 2 years follow-up and none of the patients were lost during the follow-up period. Patients’ demographic data are shown in (Table 1).

**Table 1:** Patients’ demographic data

Age	22.75 ± 3.24 (20–32)
Gender	
Male:	21 (84%)
Female:	4 (16%)
Affected shoulder joint	
Right:	15 (60%)
Left:	10 (40%)
Number of shoulder dislocation episodes	7.5 ± 3.72 (1–12)
Percentage of glenoid bone loss	22.5 ± 3.37 (12–24)
Time interval between first dislocation episode and surgery in months	5.5 ± 2.13 (3–12)
Follow-up period in months	24.25 ± 0.82 (24–26)

Values are expressed in the form of mean ± standard deviation (SD), range, number of patients, and their percentage within the group.

### Functional and radiological outcomes

**Fig. 6:**
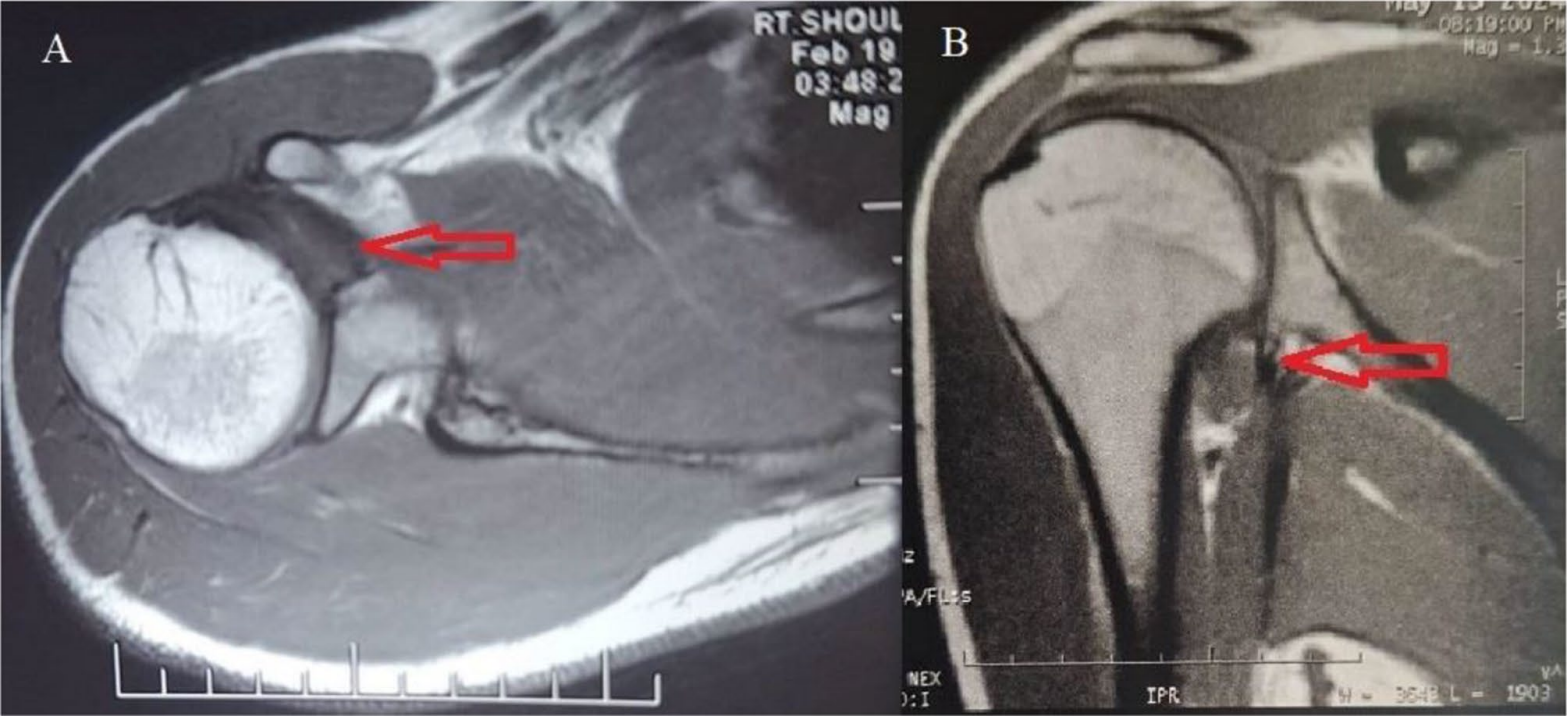
Magnetic resonance imaging showing healing of the LHB tendon to the anterior glenoid as marked by the red arrow on the (**A**) axial and (**B**) coronal slices. LHB: long head of the biceps

A statistically significant improvement in the functional scores was detected between preoperative and at 2 years follow-up. The mean Rowe score improved significantly from 21.25 ± 8.89 preoperatively to 92.5 ± 7.78 at 2 years follow-up (P-value < 0 0.001). The mean Quick DASH score showed a statistically significant amelioration from 34.62 ± 4.76 preoperatively to 14.65 ± 4.33 at 2 years follow-up (P-value < 0 0.001). Functional outcomes are shown in (Table 2). MRI was done at 6 months follow-up and showed healing of the LHB tendon to the anterior glenoid in all patients (Fig. 6).

LHB: long head of the biceps.

**Table 2:** Functional outcomes

*n* = 25	Mean ± Standard deviation (SD)	Minimum	Maximum	*p*-value
Preoperative Rowe score	21.25 ± 8.89	10	45	< 0.001
2 years follow-up Rowe score	92.5 ± 7.78*	70	100	
Preoperative Quick DASH score	34.62 ± 4.76	29.5	43.2	< 0.001
2 years follow-up Quick DASH score	14.65 ± 4.33*	9.1	20.5	

Values are expressed in the form of mean ± standard deviation (SD), minimum, and maximum. Quick DASH: Quick Disabilities of Arm, Shoulder, and Hand, n: number of patients, *:*P* < 0.05

## Complications

Two (8%) patients included in the study had recurrent shoulder dislocation during sports activities at 9 months following surgery and were treated successfully by a Latarjet procedure. The first patient had a glenoid bone loss of 12%, while the glenoid bone loss in the second patient was 16%. The first patient had a recurrence of shoulder dislocation after a contact injury during a handball match, while the second patient had a contact injury and fell to the ground on an outstretched hand during a football match. No LHB complications, neurologic or other complications were encountered in this study during the follow-up period.

## Discussion

Arthroscopic DAS can be achieved either by the onlay method as that used by Milenin and Toussaint [15], or the inlay method within a bone tunnel as done by Collin and Lädermann [11].

The onlay fixation method of LHB has been proven to produce similar healing results to the inlay fixation method [16]. In addition, the onlay technique avoids the risk of glenoid fracture due to creating large bone tunnels and has the advantage of reconstructing the labrum in cases in which Bankart repair was not possible.

The main finding in this study is that arthroscopic onlay DAS using the LHB achieved significant improvement in the functional outcomes (assessed by the Rowe and the Quick DASH scores) following treatment of recurrent AGI after a mean follow-up of 2 years. An 8% of recurrent shoulder dislocation was encountered in our study. The main outcomes in this study are comparable to various studies in the literature.

A study including 15 patients with a minimum follow-up period of 12 months showed significant amelioration of both the Western Ontario Shoulder Instability Index and Rowe scores following DAS for AGI [17]. Furthermore, only one patient (6.7% recurrence) had a recurrent shoulder dislocation and the MRI showed successful LHB healing to the anterior glenoid [17].

A retrospective study by Collin et al. evaluated the results of DAS and arthroscopic Bankart repair for recurrent anteroinferior glenohumeral instability with concomitant Subcritical GBL [18]. A significant improvement was documented between the preoperative and postoperative Rowe scores after an average follow-up period of 3.2 years and only 13.6% of patients included in the study demonstrated recurrence of shoulder dislocation [18].

A study by Wu et al. compared the functional results following arthroscopic DAS using LHB in 33 patients, and DAS using conjoined tendon in 30 patients [19]. Although a significant improvement in the postoperative functional outcomes was detected, no significant differences were detected between both groups regarding the functional scores, the return to sport, or the recurrence rate after a minimum follow-up period of 3 years [19].

Various surgical options have been used to manage Bankart lesions with concomitant GBL including the Latarjet, Bristow, and Eden-Hybinette procedures [20]. A systematic review involving forty-six studies showed that the open Bristow-Latarjet procedure is a reliable surgical choice for managing AGI [20]. Furthermore, the Eden-Hybinette procedure showed successful clinical results comparable to the Bristow-Latarjet procedure but was associated with a higher incidence of osteoarthritis and recurrence following surgery [20].

Recently, the arthroscopic technique has been put into practice in the Latarjet procedure [21]. A study including 44 patients reported successful outcomes following using the all-arthroscopic Latarjet procedure to treat recurrent shoulder dislocation [21]. However, the external rotation was significantly diminished compared to the sound shoulder, and the average complication incidence was high [21].

The anterior labrum of the shoulder joint was not repaired in the patients included in our study since it was rudimentary in chronic cases. Furthermore, the authors believe that the labroplasty effect, induced after re-routing of the LHB to the anterior glenoid rim enhances anterior glenohumeral stability. The functional outcomes were satisfactory despite not repairing the anterior labrum due to various factors. Onlay DAS theoretically enhances anterior glenohumeral stability via several mechanisms. First, is the hammock effect generated by the downward force exerted by the LHB tendon on the inferior subscapularis tendon. Second, the posteriorly directed force of the LHB on the humeral head generates the sling effect. Furthermore, the labroplasty effect, caused by placing the LHB tendon on the anterior glenoid rim theoretically enhances anterior glenohumeral stability by enlarging the contact surface area of the humeral head and glenoid.

In our study, a remplissage procedure was performed for an off-track Hill-Sachs lesion in three (12%) patients. Several studies in the literature have described the remplissage procedure in conjunction with DAS for the management of recurrent AGI with bipolar lesions [22, 23]. A recent study favored reinforcement of Bankart repair with DAS and remplissage in patients with subcritical GBL and on-track Hill-Sachs lesions, particularly in patients with high functional needs [22]. Diana-Cosmina Neculau et al. noted that DAS is an appropriate choice for the treatment of recurrent AGI in skeletally immature patients with subcritical GBL and bipolar bone injuries [23]. The remplissage procedure offers stability by resisting extreme shoulder joint external rotation, preventing the engagement of the Hill-Sachs defect, and by pulling the humeral head backward reducing the stress on the anterior soft tissue buttress [24, 25].

Patients with multidirectional instability of the shoulder joint were excluded from our study as successful results can be obtained after conservative treatment and physiotherapy programs [26]. A systematic review including twenty-four articles showed that both arthroscopic capsular plication and open capsular shift are the prime surgical operations for the management of shoulder multidirectional instability after failure of rehabilitation protocol [26]. In our study, a 2.9 mm Knotless suture anchor was used to fix the re-routed LHB tendon to the anterior glenoid. A review including ten articles reported that the advocation of either absorbable or non-absorbable anchors in the treatment of recurrent AGI was not possible and that cost-effectiveness should be considered [27].

The incidence of recurrence rate of shoulder dislocation following arthroscopic DAS in our study was 8% which was comparable to that reported by de Campos Azevedo et al. who reported a recurrence rate of 6.7%. In our study, an MRI done at 6 months follow-up showed healing of the LHB to the anterior glenoid and this was in line with several studies in the literature that reported no healing complications of LHB following DAS [17, 19].

### Limitation

The study has several limitations. First is the relatively small sample size of patients. Second is the relatively short period of postoperative follow-up. Finally, the absence of a comparative group to compare the functional outcomes for DAS versus other surgical procedures for the treatment of AGI with subcritical GBL.

## Conclusion

Dynamic anterior stabilization (DAS) using the long head of the biceps (LHB) tendon is a safe and effective procedure for the treatment of recurrent anterior shoulder instability with GBL up to 25%. DAS significantly improves shoulder functional outcomes at a minimum of 2 years follow-up.

## Data Availability

No datasets were generated or analysed during the current study.

## Abbreviations

AGI: Anterior glenohumeral instability
CT: Computerized tomography
DAS: Dynamic anterior stabilization
GBL: Glenoid bone loss
HH: Humeral head
IRB: Institutional Review Board
LHB: Long head of the biceps
MRI: Magnetic resonance imaging
SD: Standard deviation
SPSS: Statistical Package for the Social Science
SS: Subscapularis
